# Patellar Resurfacing in Total Knee Arthroplasty: A Contentious Matter

**DOI:** 10.7759/cureus.70936

**Published:** 2024-10-06

**Authors:** Prateek Upadhyay, Ankur Salwan, Kashyap Kanani, Ajay Koushik, Ankit Mittal, Saksham Goyal

**Affiliations:** 1 Orthopaedics and Trauma, Jawaharlal Nehru Medical College, Datta Meghe Institute of Higher Education and Research, Wardha, IND

**Keywords:** anterior knee pain, patellar resurfacing, patello-femoral, polyethylene, total knee arthroplasty

## Abstract

Orthopaedic surgeons hold differing opinions on replacing the patella during total knee arthroplasty (TKA) for knee osteoarthritis, sparking a continued discussion on the optimal approach. Patellar resurfacing (PR) replaces the patella's surface with a prosthetic component, aiming to enhance joint function and reduce anterior knee pain. However, it has potential complications such as avascular necrosis, fractures, and patellar clunk syndrome. This analysis evaluates the effectiveness of TKA with and without PR, highlighting the variations in outcomes between the two methods. Findings suggest that resurfacing is associated with improved clinical outcomes, activity level, patient satisfaction, and pain relief. Nevertheless, the decision should be individualized based on patient preferences and circumstances. Additional long-term randomised controlled trials incorporating outcome measures specific to patellar function are required to better understand the effects of PR versus non-resurfacing in TKA.

## Introduction and background

In total knee arthroplasty (TKA), the practice of replacing the damaged patellar joint surface with a synthetic material, typically polyethylene, remains a topic of debate among medical professionals [1]. TKA is a prevalent surgical intervention, and its prevalence continues to rise [2]. During TKA procedures, surgeons must weigh the options of preserving the natural patella or replacing it with an implant, taking into account a range of factors such as patient demographics, patellar characteristics, cartilage condition, arthritis type, imaging results, and preoperative knee symptoms [3]. In the 1980s, patellar resurfacing (PR) gained popularity but later waned due to complications such as overstuffing, polyethylene wear, osteonecrosis, fractures, extensor mechanism disruption, loosening, instability, dislocation, and patellar clunk syndrome [4-6]. Design modifications were made, and some surgeons opted against resurfacing, though non-resurfaced patients had a 10% risk of requiring subsequent resurfacing [7]. While improved techniques reduced complication rates, uncertainty about routine use persists [8].

Surgeons can be categorized as non-resurfacers, universal resurfacers, or selective resurfacers. Randomized trials have inconsistent outcomes [9]. Resurfacing is associated with favorable clinical results but carries risks of complications. The decision should be individualized based on patient factors, technique preferences, and potential risks/benefits [10]. Omitting PR in TKA may lower the risk of specific complications, but it also elevates the chance of anterior knee pain and future revision operative procedures [10].

The decision to include PR in TKA is contentious, as it seeks to mitigate severe osteoarthritis by reducing anterior knee pain and enhancing joint functionality, prompting ongoing debate [11]. However, complications such as avascular necrosis, patellar fractures, and patellar clunk syndrome emerged, prompting a review of resurfacing versus non-resurfacing outcomes [11].

## Review

Patellar resection techniques

Three main patellar resection techniques during knee replacement are using a reamer, saw guide, or freehand cutting [12]. The freehand approach involves making an osteotomy starting at the inferior patella pole, relying solely on the surgeon's experience [13]. Saw guides are challenging due to proper clamping and positioning without excessive dissection. Reamers can inadvertently cause tilting, leading to incorrect resection depth [14]. A study by Camp et al. [15] compared three resection techniques in a cohort of 90 knee replacement participants, revealing that the four-quadrant method yielded the lowest degree of patellar asymmetry post-resection (0.85 mm) (p<0.001). Additionally, the cutting guide demonstrated the highest accuracy in achieving the intended thickness (mean difference of 1.40 mm). In a separate development, Rex et al. introduced TellaMark, a guidance system designed to facilitate surgeon discretion rather than strictly dictate the cutting trajectory [12]. The goal is to regain patellar thickness and angle [11]. Complications may result from surgical technique and surgeon experience. Typical patellar thickness is 25 mm in men and 22 mm in women, with a remaining 12-15 mm bone thickness after resurfacing. Asymmetric resurfacing (>2 mm difference between medial and lateral borders) can cause difficulties [11]. Intraoperative cartilage degradation and the pre-surgery Hospital for Special Surgery patellofemoral arthritis score may suggest the need for resurfacing [16].

Significance of patella resurfacing in TKA

Anterior knee pain is a frequent postoperative complaint among TKA patients, with the patellofemoral joint being a common source of this discomfort. Understanding the causes and consequences of this issue is tough for optimizing patient outcomes and improving the effectiveness of knee replacement surgery. Waters et al. [17] performed a study in which they conducted a trial involving 514 consecutive primary press-fit condylar total knee replacements. Subjects were assigned at random to either have PR or retain their original patella. The study revealed that 25.1% of knees in the non-resurfacing group (58 out of 231 knees) reported experiencing anterior knee discomfort.

In contrast, in the knees resurfacing group, only 5.3% (13 out of 243 knees) reported the same discomfort. The group of participants who did not have resurfacing generally had lower knee scores after the surgery, and this difference was statistically significant in individuals with osteoarthritis (p<0.01). Nevertheless, there was no notable disparity in postoperative function scores between the two groups. Patients who had undergone bilateral knee replacements generally favored the side that was resurfaced.

A separate investigation conducted by Grela et al. [18] observed that PR effectively reduced the likelihood of experiencing anterior knee pain, requiring revision surgery, and encountering complications. This was observed even when patient-reported outcome measures (PROMs) were similar. In Kajino et al.'s study, rheumatoid arthritis patients underwent bilateral TKAs, with each patient randomly receiving PR on one knee. Lateral patellar release was performed in all cases to achieve optimal alignment [19]. After six years of follow-up, every patient without PR exhibited pain symptoms associated with patellofemoral dysfunction. This led to the conclusion that PR is an essential procedure in TKA, at least for rheumatoid arthritis patients.

Many studies have examined the impact of PR versus non-resurfacing on TKA. He et al. [20], Fu et al. [21], and Li et al. [22] found an increased risk of revision with non-resurfacing, while Lindstrand et al. [23] observed no difference in revision rates between the two approaches. An investigation by Patil et al. [24] involving PR, non-resurfacing, and patelloflex groups showed improved Knee Society scores and patient satisfaction with resurfacing.

Pilling et al. [25] performed a comparison between two procedures. The results showed that 13% of resurfaced knees and 24% of non-resurfaced knees experienced anterior knee pain. The level of patient satisfaction was 90% for resurfacing and 89% for the non-resurfacing procedure. Nevertheless, a random-effects model demonstrated a statistically significant increase in patellofemoral issues among the non-resurfacing group (p=0.02). In a comprehensive analysis conducted by Grassi et al. [26], it was shown that PR resulted in lower reoperation rates at the one to two-year follow-up in 20 randomized controlled trials (RCTs).

Several studies have examined the PR in TKA. Tang et al. [27] suggested that resurfacing can improve clinical scores and reduce reoperation rates over a one to two-year follow-up in patients with knee osteoarthritis. Pavlou et al. [28] observed that secondary PR might help address anterior knee pain after the initial arthroplasty surgery, potentially explaining higher reoperation rates in non-resurfaced cases. However, a study by Aunan et al. [29] reported that, while PR improved knee injury and osteoarthritis outcome scores, it did not significantly impact Knee Society Scores, Oxford Knee Scores, or visual analog scale measures.

Considering the challenge of accurately positioning the replacement button during a subsequent replacement, which is most technically sound to resurface the patella. This analysis only weakly indicates the need for further patella resurfacing, as concluded by a comprehensive assessment conducted by van Jonbergen et al. [30]. Simultaneously, 64% of subjects had enhanced efficient ratings, but there was no amelioration in clinical scores following secondary replacement, and complications were documented. Complications such as infections, delayed wound healing, patellar instability, and fractures have been well-documented [30]. Moreover, a study conducted by Thomas et al. [31] on the Trent and Wales Arthroplasty Registry observed that 44% of participants experienced positive outcomes after undergoing patella resurfacing following their initial knee arthroscopy. The results indicated that patella resurfacing should only be contemplated if there are substantial alternative diagnoses. Zmistowski et al. [32] observed that patella resurfacing resulted in a slight enhancement in quality-adjusted life years (QALY) for selective and indiscriminate patella resurfacing.

Conversely, a study by Gerow et al. [33] reports that PR based on patellofemoral joint (PFJ) degeneration and mechanical PFJ findings show equivalent lead in PROMs for resurfaced and unresurfaced knees. Sato et al. [34] examined patellar status using MRI imaging to provide another perspective on patellofemoral joint progression in the TKA without PR. Over 15 years (2009-2014), 40 patients and 59 knees were studied. The patient received TKA with zirconia ceramic femoral component without PR. Annual radiological and T2-weighted MRI evaluations were done for five years. For better evolution standardization, patellar cartilage was separated into medial, central, and lateral zones. The Japanese Oxford Score and American Knee Society Score were used to evaluate patients. A notable increase in lateral shift ratio was observed, rising from 7.1% to 14.6%, accompanied by a significant decline in patellar cartilage thickness (p<0.05). This deterioration was consistent across all three zones, with cartilage thickness ultimately reducing to less than half its original value. Furthermore, clinical outcomes at the five-year mark were substantially lower than those at one year postsurgery, indicating a decline in patient well-being over time.

Patellar prosthesis reintervention was needed for four individuals. In five years, patellar non-resurfacing in TKA will reduce patellar cartilage thickness by greater than 50%, causing femoropatellar dysfunction and needing patellar prosthesis reintervention. Burnett et al. [35] performed a randomized trial with cruciate retaining (CR) prosthesis on 32 participants (64 knees) for bilateral TKA. Patients were randomly assigned to subgroups with and without PR and followed for 10 years. The Knee Society Clinical Rating Score (KSCRS, max 200 points) was used to evaluate patients. Patient satisfaction, nonunion discomfort, or revision rate between the two groups. Reoperation for femoropatellar complications was required for two subjects (7.4%) in the non-resurfaced group and one subject (3.5%) in the resurfaced group. While debatable, the clinical outcomes of TKA were considered comparable with or without PR.

Another prospective, double-blind study of 220 TKA knees was undertaken by Wood et al. [8]. A 48-month follow-up study examined the outcomes of 220 knees, with 128 undergoing TKA without PR and 92 with resurfacing. The study utilized the Stair Climbing Rising Score evaluation tool and a stairclimbing test to assess patient outcomes. While the reoperation rates for femoropatellar issues were comparable between the two groups (12% vs. 10%, p=0.650), the non-resurfaced group demonstrated significantly higher levels of anterior knee pain (p=0.016), indicating a notable difference in patient experiences.

Thus, at the last follow-up, 39 (31%) of the 128 knees without PR reported anterior knee pain, while 15 (16%) of the 92 with resurfacing did. The author believes patellar non-resurfacing will cause pain, joint dysfunction, and subject dissatisfaction.

During a six-month follow-up study, Kaseb et al. [36] found that there was no notable disparity in observations between patella resurfacing and non-resurfacing in TKA. In a study conducted by Gupta et al. [37], it was found that both groups of subjects who underwent knee replacement surgery, with and without PR, experienced significant improvements in functional outcomes, pain relief, and clinical outcomes. Both cohorts exhibited subject satisfaction levels exceeding 90%, and there was no statistically significant disparity in Knee Society scores (p=0.185), patient contentment (p=0.467), or pain relief (p=0.215) between the two groups. In a study of 158 participants and 162 knees who underwent TKA, De Campos Júnior noted anterior knee pain in patellar non-resurfacing. The patients were randomized into two equal categories, PR or not [38]. Using Lequesne and WOMAC ratings, clinical and functional outcomes were examined over five years. While the study found no significant differences in various outcome measures, including Lequesne scores, WOMAC overall scores, stiffness, and functional capacity, a notable exception emerged in the WOMAC pain subgroup. The resurfaced group exhibited significantly lower pain scores (p=0.036), highlighting the benefit of PR in lowering pain. Despite similar joint function outcomes between groups, the results suggest that patellar non-resurfacing may compromise patients' quality of life due to increased anterior knee pain.

Feller et al. [39] performed an RCT on 40 patients with mild patella deformities and osteoarthritis who underwent primary TKA performed by a surgeon using a standardized prosthesis. Participants were randomly divided into two groups: one where the patella was retained in its natural state and another where it was resurfaced with a cemented, all-polyethylene component, regardless of the condition of the patellar articular cartilage.

No surgery was performed on retained patellae save osteophyte excision. At three years, the Hospital for Special Surgery (HSS) knee score and a new 30-point patellar score were used to evaluate all 38 survivors. There was no TKA revision, although two resurfacing patients underwent an unrelated operation. For patellar retention, the mean HSS and patellar scores were 89 and 28 at follow-up and, for resurfacing, 83 and 26. Women and heavier patients had significantly lower scores. The retention group was substantially superior at stairclimbing. Although PR had minimal problems, we found no medium-term benefit from it with TKA for osteoarthritis if the patella was not substantially damaged. Another analysis by Parsons et al. [40] reported patella resurfacing to be a cost-effective procedure. However, this study found the participants' reported outcomes for resurfacing and non-resurfacing to be inconclusive. An in-depth examination of the existing literature indicates that the difficulties associated with patella resurfacing are outdated, as they have been reduced because of advancements in implant design and surgical technique. Long-term cost-effectiveness was achieved through routine resurfacing, resulting in potential savings of £104 per case. Additionally, the procedure demonstrated a 4% absolute risk decrease in rates of revision. Ultimately, the exercise of surgical judgment in the process of selective resurfacing proved susceptible to errors.

Conversely, Maniar et al. [41] investigated the incidence and underlying reasons for secondary PR (SPR) following TKA using a contemporary TKA design. The findings suggest that routine PR during primary TKA may be beneficial in reducing the likelihood of subsequent surgical interventions. It also recommends reevaluating the notion that delayed SPR does not enhance clinical results.

Vukadin et al. [42] randomized patients to either have patellar surface replacement during TKA or receive a patellar surface replacement without undergoing this procedure. The study involved 60 patients. A comparative study involving 60 patients undergoing TKA revealed no significant differences in various analyzed characteristics between the two groups: 30 patients who received TKA with PR (TKAPR) and 30 patients who underwent TKA without PR (TKA without PR). However, a notable exception emerged at the six-month follow-up, where the Oxford Knee Score significantly favored the TKAPR group, indicating improved knee function and patient outcomes.

Currently, as per this study, the surgeon has the sole authority to decide whether or not to replace the patella. Thus, it could be beneficial to establish criteria for people who would benefit from patella resurfacing for both patients and orthopedic surgeons conducting complete knee replacements. While this study's data did not conclusively support the need for patella resurfacing in valgus deformity patients, the patella resurfacing group's outcomes were marginally better, and this tendency might continue if a larger patient series was used. In another study by Bourne et al. [43], in an investigation, 100 patients with osteoarthritic knees underwent identical total knee replacement procedures, with 50 receiving patella resurfacing and 50 without. Preoperative assessments were conducted, followed by evaluations at least two years post-operation using disease-specific and functional capacity outcome measures. Notably, two participants in the non-resurfacing group needed reoperation due to anterior knee pain. At the two-year mark, the non-resurfacing group reported significantly less pain and demonstrated improved knee flexor torques compared to the resurfacing group. However, both groups showed similar results in knee society function scores, 30-second stair climbing, and knee extensor torques.

Tang et al. [44] in their review of 50 studies encompassing 5,586 knees. They noticed notable decreases in patellar revision rate (RR: 0.41, 95% CI: 0.19, 0.88; p=0.02; I²=24.20%) and non-patellar revision rate (RR: 0.64, 95% CI: 0.55, 0.75; p<0.001; I²=0%) following PR. PR markedly decreased the incidence of anterior knee pain compared to non-resurfacing (RR: 0.72, 95% CI: 0.57, 0.91; p=0.006; I²=69.5%). PR led to a markedly reduced incidence of patellar clunk (RR: 0.58, 95% CI: 0.38, 0.88; p=0.01; I²=0%), an enhanced patellar score (MD: 1.24, 95% CI: 0.67, 0.81; p<0.001; I²=73.8%), but an extended operative duration (MD: 8.59, 95% CI: 5.27, 11.91; p<0.001; I²=88.8%).

Adam et al. [11], in their case report and their literature review, advised the resurfacing of the patella in TKA and the need to formulate a generally applicable protocol for patella resurfacing in TKA.

Mayman et al. [45] conducted a study among a total of hundred study participants with osteoarthritic knees who were randomly assigned to either undergo patella resurfacing or not have their patella resurfaced. Each patient had treatment using a singular prosthesis that included an anatomically engineered patellofemoral articulation. Out of the patients who did not have their joint surface resurfaced, two of them needed to undergo another surgery due to patellofemoral complications. Among patients with the resurfaced patella, only one individual required revision surgery due to patellofemoral issues. Over an 8-10-year follow-up period, the Knee Society Clinical Ratings scores showed no significant disparities between the resurfaced and non-resurfaced groups. However, the resurfaced group demonstrated a marked reduction in anterior knee pain during weight-bearing activities such as walking and stair climbing. Furthermore, patient satisfaction levels were significantly higher in the resurfaced group, with 80% reporting high satisfaction in their TKA outcome, in spite of 48% in the non-resurfaced group. In a nutshell, the determination regarding resurfacing should be taken by considering patient preferences and unique circumstances on a case-by-case basis.

## Conclusions

TKR surgery that involves resurfacing the back of the kneecap (patella) appears to provide benefits such as improved outcomes, functionality, patient contentment, and pain relief for those undergoing the procedure for knee osteoarthritis. A review of studies found a notably higher rate of anterior knee pain after knee replacement without patellar resurfacing. As such, it may be advisable to consider patellar resurfacing during a total knee replacement if feasible. However, research findings are conflicting on whether patella resurfacing leads to meaningfully better results compared to not resurfacing the patella. Ultimately, the decision to resurface is made on a case-by-case basis, taking into account patient preferences and individual circumstances. Further long-term randomized studies using patella-specific outcome measures are needed to evaluate better newer implant designs that allow for patellar resurfacing. In summary, while patellar resurfacing may reduce anterior knee pain, its overall superiority is unclear, so shared decision-making is warranted.
